# Extracellular volume fraction quantification by equilibrium contrast-enhanced CT via automated segmentation predicts survival outcomes in bladder cancer: a propensity score-matched study

**DOI:** 10.1080/07853890.2025.2534856

**Published:** 2025-07-21

**Authors:** Weiwei Liu, Yusheng Guo, Bingxin Gong, Xiaona Fu, Jie Lou, Peng Sun, Yi Li, Shanshan Jiang, Lianwei Miao, Feng Pan, Lian Yang

**Affiliations:** a Department of Radiology, Union Hospital, Tongji Medical College, Huazhong University of Science and Technology, Wuhan, China; b Hubei Provincial Clinical Research Center for Precision Radiology & Interventional Medicine, Wuhan, China; c Hubei Key Laboratory of Molecular Imaging, Wuhan, China; d Clinical & Technical Support, Philips Healthcare, Beijing, China

**Keywords:** Extracellular volume fraction (ECV), bladder cancer (BLCA), propensity score matching (PSM), prognostic biomarker, recurrence-free survival (RFS)

## Abstract

**Background:**

Extracellular volume fraction (ECV) is the unoccupied space or volume of tissue that is not occupied by cells and can be used to assess the growth and invasive behavior of solid tumors. Our study aimed to investigate the ability of extracellular volume fraction in predicting the prognosis of BLCA patients.

**Materials and methods:**

A retrospective study recruited 319 BLCA patients who underwent surgery. The average CT value was recorded by creating 3D masks of the tumor and blood vessels, which was then used to calculate the preoperative ECV. A propensity score matching (PSM) was performed at a ratio of 1:1 to balance the baseline characteristics between the two groups. Correlations between ECV scores and pathological T stage (whether muscle invasion was present) were determined by using Pearson correlation coefficients. The effects of clinical or pathologic prognostic factors and ECV on recurrence-free survival (RFS), overall survival (OS) were assessed by univariate and multivariate analyses using Cox proportional risk models.

**Results:**

The 319 patients were divided into two groups, ECV-lower group (*n* = 178) and ECV-higher group (*n* = 141). After PSM analysis, patients in the ECV-lower group (*n* = 121) and patients in the ECV-higher group (*n* = 141) were obtained. In multivariate analysis, ECV was the independent prognostic factor for RFS (ECV-higher: HR: 2.55, 95% Cl: 1.85 to 3.51, *p* < 0.01), and for OS (ECV-higher: HR: 5.46, 95% Cl, 1.89 to 15.73, *p* = 0.02). A positive correlation between ECV and pathologic T stage was found both before and after PSM (*r* = 0.225, *p* < 0.001; *r* = 0.136, *p* = 0.028).

**Conclusion:**

ECV can be used as a noninvasive biomarker to assist in the prognostic assessment of BLCA patients. Higher preoperative ECV indicates a poor prognosis for BLCA and requires aggressive treatment.

## Introduction

Bladder cancer (BLCA) is the 10th most common cancer globally and the 4th most frequently diagnosed cancer in men [1]. Radical cystectomy (RC) remains the gold standard treatment for patients with high-risk non-muscle invasive and muscle invasive urothelial carcinoma of the bladder (UCB) [2], however, despite receiving this aggressive local treatment, the postoperative survival rates of patients are still significantly affected by pathological stage [3]. Studies have shown that patients with locally advanced disease after RC have a 5-year survival rate of 26%–64% [4]. This prognostic difference reflects the high degree of heterogeneity in the treatment of BLCA and suggests the importance of risk stratification and individualized treatment regimens.

The high degree of tumor heterogeneity, drug resistance and microenvironmental complexity are key factors influencing treatment outcomes [5]. For example, single-cell sequencing techniques revealed the presence of anti-apoptotic tumor subpopulations in bladder cancer, which interact with the immune microenvironment to promote malignant progression through activation of signalling pathways such as FGFR and CXCL [6]. In addition, stromal cells in the tumor microenvironment (e.g. myofibroblast carcinoma-associated fibroblasts MCAFs and inflammatory carcinoma-associated fibroblasts) modulate immunosuppression in the tumor microenvironment through different mechanisms [7], further adding to therapeutic complexity. In recent years, the application of prognostic models based on molecular markers (e.g. Ascore, 5-gene risk model) and liquid biopsy techniques (e.g. circulating tumor cell [CTC], circulating endothelial cell [CEC]) has significantly improved prediction accuracy [8]; however, most existing prognostic assessment tools rely on invasive procedures (e.g. biopsy) or clinicopathologic data [9–11]. Therefore, there is an urgent need for the discovery of new, easily accessible biomarkers to more accurately predict the prognosis of patients with BLCA.

ECV is a quantitative biomarker calculated using hematocrit (Hct), Hounsfield Unit (HU) values of tumors and vessels in the plain and contrast equilibrium phases, representing the sum of extravascular-extracellular and intravascular interstitial spaces, the former of which expands with the progression of tissue fibrosis, not only can be used to assess organ fibrosis (e.g. cardiac, pancreatic, liver) [12–14], but can also be applied to evaluate the extracellular matrix volume in solid tumors (such as pancreatic cancer and renal cancer) [15,16], thereby assessing tumor growth and invasive behavior. Although ECV has been validated as an effective prognostic indicator in other types of tumors, its application in BLCA is still in its infancy, with a paucity of relevant studies, and more data are urgently needed to validate its clinical value. Therefore, this study aims to explore the potential of ECV in preoperative prognostic assessment for BLCA patients, to evaluate the relationship between ECV and patients’ recurrence-free survival (RFS) and overall survival (OS), and to further compare ECV with conventional pathologic staging and molecular markers in prognostic prediction. This study is expected to provide a new and reliable non-invasive predictive tool for clinical decision-making in bladder cancer, and to provide a basis for the development of individualized treatment plans.

## Materials and methods

This study followed the principles of the Helsinki Declaration and was sanctioned by the Ethics Committee of Tongji Medical College, Huazhong University of Science and Technology. This retrospective cohort study has been approved by the local ethics committee and the institutional review board of Tongji Medical College (Institutional Review Board No. S187), and they have waived the need for informed consent. Researchers analyzed the clinical data anonymously and retrospectively.

### Study design and population

**Figure 1.**
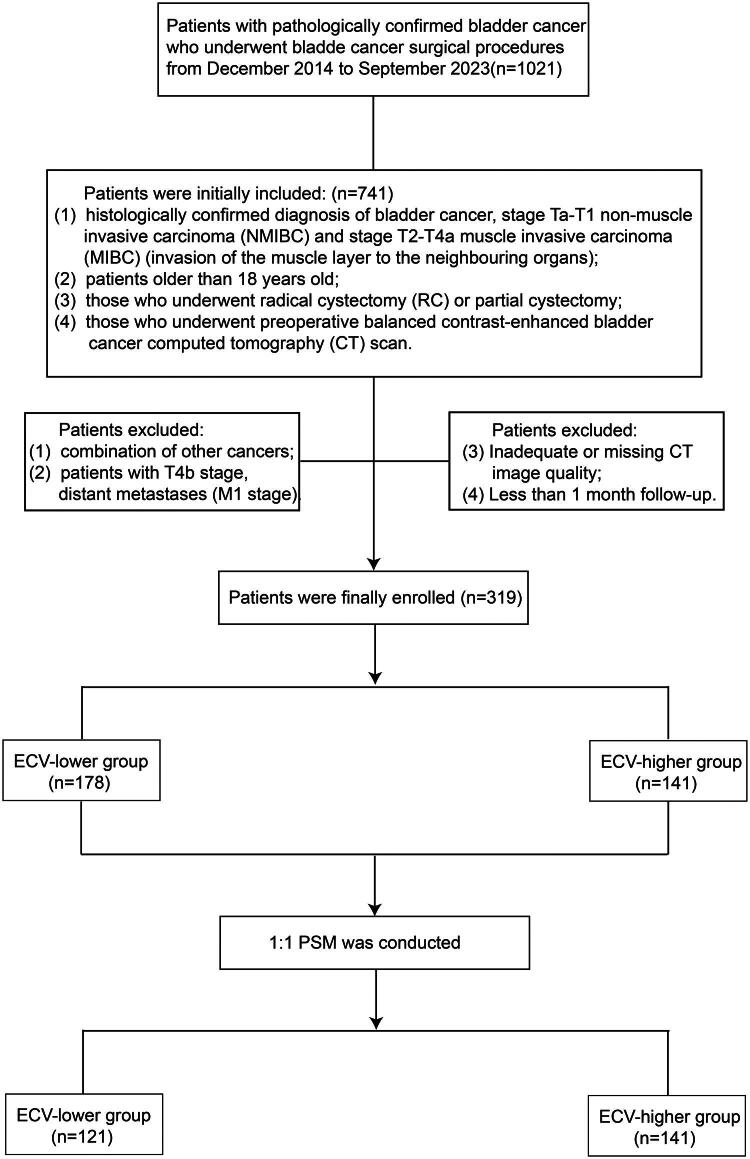
Patient flowchart.

This study included a cohort of bladder cancer patients who underwent bladder tumor surgery at our hospital from August 2019 to March 2023. The inclusion criteria were as follows (1) histologically confirmed diagnosis of bladder cancer, stage Ta-T1 non-muscle invasive carcinoma (NMIBC) and stage T2-T4a muscle invasive carcinoma (MIBC) (invasion of the muscle layer to the neighbouring organs); (2) patients older than 18 years old; (3) those who underwent radical cystectomy (RC) or partial cystectomy (4) those who underwent preoperative balanced contrast-enhanced bladder cancer computed tomography (CT) scan. Exclusion criteria were (1) combination of other cancers; (2) patients with T4b stage, distant metastases (M1 stage). (3) Inadequate or missing CT image quality; (4) Less than 1 month follow-up. The flowchart for recruiting patients is shown in Figure 1.

A total of 319 bladder cancer patients were ultimately included in the study. Preoperative patient demographics, pathology, and laboratory information were recorded. Demographics included age, gender, drinking, smoking, hypertension, diabetes, and chemotherapy. Pathologic information included pathologic tumor grade, pathologic stage, and whether muscle invasion was present. Laboratory variables including hematocrit levels, neutrophils, lymphocytes, platelets, albumin, and globulin were measured within one week prior to CT. Propensity score matching (PSM) in a 1:1 fashion between pathologic T stage < T2 (non-muscle-invasive bladder cancer, NMIBC) and ≥ T2 groups (muscle-invasive bladder cancer, MIBC).

We define past history as follows: Hypertension: systolic blood pressure ≥140 mmHg or diastolic blood pressure ≥90 mmHg, history of doctor-diagnosed hypertension or use of antihypertensive medication. Diabetes: fasting blood glucose ≥7.0 mmol/L, history of doctor-diagnosed diabetes or use of hypoglycaemic drugs [17]. Cardiovascular disease: myocardial infarction; or stroke or angina, or both.

During the follow-up period, the primary endpoint was RFS, and the secondary endpoint was OS. RFS is the time from the end of treatment to the first tumour recurrence or death from any cause after the patient has been treated surgically to achieve complete remission (CR). OS is defined as the time from the first surgery to the patient’s death from any cause. Follow-up assessment: RFS is based on CT images and clinical examination, OS is based on clinical history and telephone follow-up.

### CT imaging technology

Contrast-enhanced CT was performed preoperatively in our hospital using three CT scanners (IQon CT; Definition AS+; SOMATOM ForceCT), and the scanning parameters are shown in Supplementary Table 1. A single intraventricular mass injection of iodine-containing contrast agent was used (300–350mg/ml, flow rate 2 ∼ 3 ml/s, dosage 60.0 ∼ 80.0).

Non-contrast CT scan images and equilibrium-phase CT images were obtained for each patient. Equilibrium Phase refers to a time window of 3–5 min after intravenous injection of extracellular iodinated contrast agent. At this point the contrast agent is in dynamic equilibrium between the intravascular (plasma) and extravascular extracellular space (e.g. tissue interstitium), i.e. the concentration of contrast agent in plasma is approximately equal to that in the tissue extracellular space and the rate of transvascular exchange is stable, and the ratio of tissue and iodine concentration ratio in the blood pool directly reflects the extracellular volume ratio [18,19] (Supplementary Figure 1). The scanning area was scanned from the upper pole of the kidney to the lower edge of the pubic symphysis.

**Figure 2.**
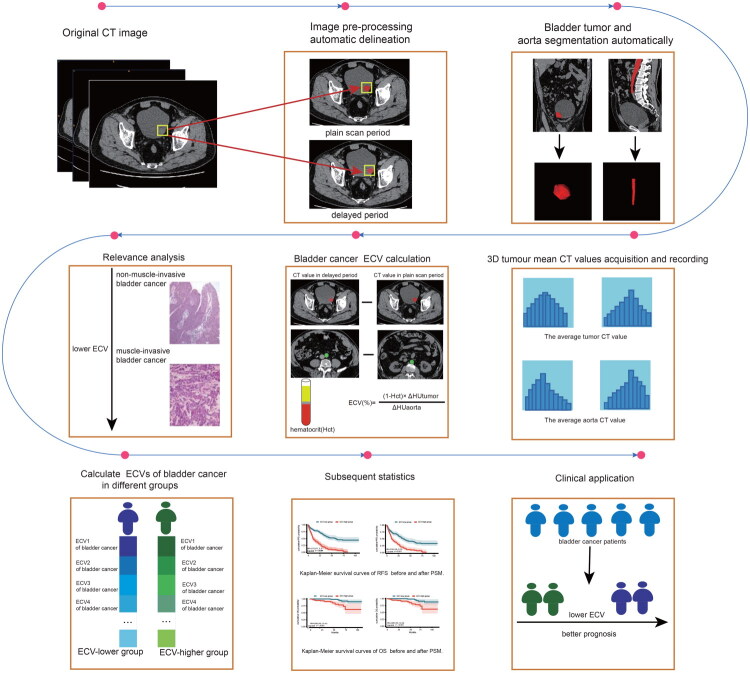
Flow chart of region of interest delineation for bladder cancer and vessels.

The entire region of bladder cancer and the abdominal aorta were automatically delineated using the no-new-Net (nnU-Net) models (in-house trained model for bladder cancer and pre-trained Task017_AbdominalOrganSegmentation model for abdominal aorta) (10.1038/s41592-020-01008-z) (Figure 2), followed by manual calibration of the contours of the bladder cancer and abdominal aorta, and the average HU values of the tumor and blood vessels were obtained using ITK-SNAP software (https://www.itksnap.org/) (Supplementary Figure 2). Bladder cancer is configured according to the profile structure of nnUNetv2 (source code publicly available on GitHub: https://github.com/MIC-DKFZ/nnUNet/blob/master/documentation/how_to_use_nnunet.md), after which it undergoes preprocessing, including normalisation, resampling and other operations, and data attributes (e.g. image size, resolution, intensity distribution) are automatically analysed and 2DU-Net configurations, 3D full resolution U-Net configurations are generated. Five-fold cross-validation is used to train 2DU-Net configuration, 3D full-resolution U-Net configuration for model training. Finally, the results of 3D full-resolution U-Net configuration training were used for dataset inference in this study. The Dice coefficients for both the flat sweep and delay periods were obtained to be greater than 0.85 (0.88,0.86). All regions of interest (ROIs) were reviewed and adjusted by the radiologist (F.Q.Q, >10 years of experience in diagnostic imaging) using ITK snap to ensure accuracy.

Calculate the ECV score for bladder cancer using the formula provided:

ECV fraction (%) = (1 − hematocrit) × (ΔHU_tumor_/ΔHU_aorta_) × 100, respectively, where ΔHU_tumor_ and ΔHU_aorta_ are HU in equilibrium phase minus HU before contrast agent administration of the tumor and the aorta, respectively.

### Statistical analysis

Continuous variables are expressed as mean with standard deviation or median with interquartile range (IQR). Categorical variables are represented by numbers with percentages. Student’s *t*-test was used for normally distributed data and Mann–Whitney *U*-test for non-normally distributed data. The optimal critical value of ECV relative to RFS was calculated using the survminer package (surv_cutpoint algorithm) of the R software and accordingly the patients were categorized into ECV-higher group (a group with lower ECV) and ECV-lower group (a group with higher ECV), and the clinicopathological characteristics and RFS of the two groups were compared. In this study, PSM was performed to minimize selection bias between ECV-higher group and ECV-lower group. Matching propensity scores 1:1 using the nearest neighbor method with a width of 0.02 caliper. Correlations between outcomes and variables were examined using Kaplan-Meier survival curves, and results were expressed as hazard ratios (HR) and 95% confidence intervals (CI). One-way Cox regression analysis was used to evaluate the correlation of clinical factors, morphology and ECV with RFS and OS. All statistical analyses were performed using SPSS version 25 (SPSS Inc, Chicago, IL, USA) and R (version 4.1.2; https://www.r-project.org/), and *p* < 0.05 was considered statistically significant.

## Results

### Baseline characteristics of patients

ECV scores from automatic segmentation based on the nnU-Net model and manual segmentation by radiologists were in good agreement, with an intragroup correlation coefficient (ICC) of 0.972 (95% confidence interval: 0.965–0.977). The agreement between the two methods was further verified by Bland-Altman analysis: the bias in ECV scores was small (3.4%, solid line) and the 95% consistency limit (dashed line) ranged from −9.8% to 12.6% (Supplementary Figure 3). A total of 319 BLCA patients who underwent surgical treatment were included, with 178 patients in the ECV-lower group (*n* = 178) and 141 patients in the ECV-higher group (*n* = 141). Table 1 presents the demographic and clinical characteristics of patients at baseline, both before and after PSM analysis. Before PSM, the ECV-low group had a higher proportion of patients with non-muscle invasive disease (80.3% vs. 62.3%, *p* < 0.001), and a higher proportion of patients with preoperative chemotherapy (62.9% vs. 51.1%, *p* = 0.033). After PSM, the distribution of baseline characteristics was balanced between the two groups, with no significant differences observed.

**Table 1.** Baseline characteristics of patients before and after PSM analysis.

	Before PSM			After PSM		
Characteristics	ECV-lower	ECV-higher	*P* value	ECV-lower	ECV-higher	*P* value
Patient characteristics						
Patients, *n*	178	141		121	141	
Gender, *n* (%)			0.505			0.708
Male	149 (83.7%)	114 (80.9%)		100 (82.6%)	114 (80.9%)	
Female	29 (16.3%)	27 (19.1%)		21 (17.4%)	27 (19.1%)	
Age, no. (%)			0.553			0.667
<65	90 (50.6%)	76 (53.9%)		62 (51.2%)	76 (53.9%)	
≥65	88 (49.4%)	65 (46.1%)		59 (48.8%)	65 (46.1%)	
Pathologic T stage, *n* (%)			<0.001			0.137
<T2	35 (19.7%)	52 (37.7%)		86 (71.1%)	86 (62.3%)	
≥T2	143 (80.3%)	86 (62.3%)		35 (28.9%)	52 (37.7%)	
Body mass index, *n* (%)			0.429			0.520
<Median	87 (52.7%)	64 (48.1%)		58 (52.3%)	64 (48.1%)	
≥Median	78 (47.3%)	69 (51.9%)		53 (47.7%)	69 (51.9%)	
Pathologic tumor grade, *n* (%)			0.064			0.479
Low grade/PUNLMP	87 (49.4%)	55 (39%)		52 (43.3%)	55 (39%)	
High grade	89 (50.6%)	86 (61%)		68 (56.7%)	86 (61%)	
NLR, *n* (%)			0.051			0.124
≤2	65 (36.5%)	37 (26.2%)		41 (33.9%)	35 (25.2%)	
>2	113 (63.5%)	104 (73.8%)		80 (66.1%)	104 (74.8%)	
PLR, *n* (%)			0.058			0.256
≤150	117 (65.7%)	78 (55.3%)		74 (61.7%)	76 (54.7%)	
>150	61 (34.3%)	63 (44.7%)		46 (38.3%)	63 (45.3%)	
Adjuvant chemotherapy, *n* (%)			0.033			0.101
Yes	112 (62.9%)	72 (51.1%)		74 (61.2%)	72 (51.1%)	
No	66 (37.1%)	69 (48.9%)		47 (38.8%)	69 (48.9%)	
Hypertension, *n*(%)			0.650			0.281
Yes	29 (29.6%)	33 (26.8%)		40 (33.1%)	38 (27%)	
No	69 (70.4%)	90 (73.2%)		81 (66.9%)	103 (73%)	
Diabetes, *n* (%)			0.087			0.055
Yes	31 (17.4%)	15 (10.6%)		23 (19%)	15 (10.6%)	
No	147 (82.6%)	126 (89.4%)		98 (81%)	126 (89.4%)	
Drinking, *n* (%)			0.655			0.337
Yes	39 (21.9%)	28 (19.9%)		30 (24.8%)	28 (19.9%)	
No	139 (78.1%)	113 (80.1%)		91 (75.2%)	113 (80.1%)	
Cardiovascular disease, *n* (%)			0.304			0.301
Yes	20 (11.2%)	11 (7.8%)		14 (11.6%)	11 (7.8%)	
No	158 (88.8%)	130 (92.2%)		107 (88.4%)	130 (92.2%)	
Smoking, *n* (%)			0.182			0.103
Yes	59 (33.1%)	37 (26.2%)		43 (35.5%)	37 (26.2%)	
No	119 (66.9%)	104 (73.8%)		78 (64.5%)	104 (73.8%)	

ECV: extracellular volume fraction; SD: standard deviation; NLR: neutrophil-to-lymphocyte ratio; PLR: platelet-to-lymphocyte ratio; A/G ratio: albumin to globulin ratio; BMI: body mass index; PUNLMP: papillary urothelial neoplasms of low malignant potential.

### Correlation analysis

There was a positive correlation between ECV and muscle invasion status both before and after PSM (*r* = 0.225, *p* < 0.001; *r* = 0.136, *p* = 0.028). BLCA patients with muscle invasion exhibited higher ECV scores, which was statistically significant.

### Optimal cutoff value for ECV

A statistically significant difference in RFS was noted between the two groups when the optimal ECV cutoff value was set at 42.05 (Supplementary Figure 4). Based on this cutoff, patients were categorized into the ECV-lower group and ECV-higher group.

### Survival analysis

**Figure 3.**
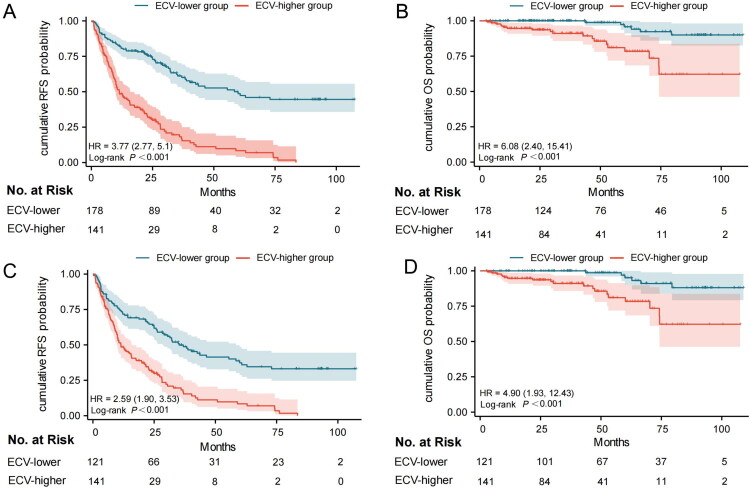
Kaplan–Meier survival curves of OS and RFS among BLCA patients before and after PSM.

Before PSM, log-rank tests indicated that patients in the ECV-higher group had shorter RFS (*p* < 0.001, median RFS for ECV-lower: 59.9 months and ECV-higher: 11.4 months, Figure 3A) and shorter OS (*p* < 0.001, median OS for both groups could not be obtained, Figure 3B). Similarly, after PSM, log-rank tests indicated that patients in the ECV-higher group had shorter RFS (*p* < 0.001, median RFS for ECV-lower: 35.2 months and ECV-higher: 11.4 months, Figure 3C) and shorter OS (*p* < 0.001, median OS for both groups could not be obtained, Figure 3D).

### Cox regression analysis and subgroup analysis

Before PSM, univariate regression analysis identified ECV, pathologic tumor grade, pathological T stage, adjuvant chemotherapy, and neutrophil-to-lymphocyte ratio (NLR) as potential predictors of RFS. And ECV, pathologic tumor grade, pathological T stage, age, adjuvant chemotherapy, and NLR were identified as potential predictors of OS. These covariates were subsequently incorporated into the multivariate regression analysis. In multivariate analysis, ECV-higher group (HR, 3.34 [95% Cl, 2.42 to 4.60]; *p* < 0.001), pathological T stage ≥ T2 (HR, 3.48 [95% Cl, 2.36 to 5.13]; *p* < 0.001) were significantly associated with shorter RFS (Supplementary Table 2). ECV-higher group (HR, 6.08 [95% Cl, 2.10 to 17.61]; *p* < 0.001), age ≥65 (HR, 7.44 [95% Cl, 2.41 to 22.96]; *p* < 0.001), and without adjuvant chemotherapy (HR, 3.15 [95% Cl, 1.18 to 8.39]; *p* = 0.022) were significantly correlated with shorter OS (Supplementary Table 3).

After PSM analysis, ECV-higher group (HR, 2.55 [95% Cl, 1.85 to 3.51]; *p* < 0.001) and pathological T stage ≥ T2 (HR, 2.92 [95% Cl, 2.01 to 4.25]; *p* < 0.001) were significant risk factors associated with shorter RFS (Table 2), and ECV-higher group (HR, 5.46 [95% Cl, 1.89 to 15.73]; *p* = 0.002), and age ≥65 (HR, 7.36 [95% Cl, 2.38 to 22.72]; *p* < 0.001), and without adjuvant chemotherapy (HR, 3.02 [95% Cl, 1.22 to 7.46]; *p* = 0.017) were significant risk factors associated with shorter OS (Table 3).

**Table 2.** Cox proportional hazards analyses for RFS after PSM analysis.

Parameter	Univariate analysis		Multivariate analysis	
	Hazard ratio (95% CI)	*P* value	Hazard ratio (95% CI)	*P* value
Gender				
Male	Reference			
Female	0.972 (0.663–1.424)	0.883		
Age				
<65	Reference			
≥65	0.860 (0.644–1.150)	0.310		
Pathologic T stage				
<T2	Reference		Reference	
≥T2	2.857 (2.088–3.908)	<0.001	2.920 (2.007–4.249)	<0.001
Pathologic tumor grade				
Low grade/PUNLMP	Reference		Reference	
High grade	1.484 (1.097–2.007)	0.010	0.895 (0.623–1.287)	0.550
Adjuvant chemotherapy				
No	Reference		Reference	
Yes	0.629 (0.469–0.843)	0.002	0.797 (0.589–1.078)	0.141
Body mass index				
<Median	Reference			
≥Median	1.058 (0.783–1.430)	0.712		
Cardiovascular disease				
No	Reference			
Yes	0.722 (0.418–1.246)	0.242		
Hypertension				
No	Reference			
Yes	0.871 (0.629–1.207)	0.407		
Diabetes				
No	Reference			
Yes	0.750 (0.484–1.161)	0.197		
Drinking				
No	Reference			
Yes	0.927 (0.659–1.305)	0.663		
Smoking				
No	Reference			
Yes	0.850 (0.621–1.163)	0.309		
NLR				
≤2	Reference			
>2	1.163 (0.836–1.616)	0.371		
PLR				
≤150	Reference			
>150	1.121 (0.835–1.506)	0.447		
Group				
ECV-lower	Reference		Reference	
ECV-higher	2.586 (1.896–3.528)	<0.001	2.549 (1.853–3.508)	<0.001

ECV: extracellular volume fraction; SD: standard deviation; NLR: neutrophil-to-lymphocyte ratio; PLR: platelet-to-lymphocyte ratio; A/G ratio: albumin to globulin ratio; BMI: body mass index; PUNLMP: Papillary Urothelial Neoplasms of Low Malignant Potential.

**Table 3.** Cox proportional hazards analyses for OS after PSM analysis.

Parameter	Univariate analysis		Multivariate analysis	
	Hazard ratio (95% CI)	*P* value	Hazard ratio (95% CI)	*P* value
Gender				
Male	Reference			
Female	0.970 (0.364–2.588)	0.951		
Age				
<65	Reference		Reference	
≥65	4.544 (1.705–12.113)	0.002	6.223 (2.268–17.073)	<0.001
Pathologic T stage				
<T2	Reference		Reference	
≥T2	3.135 (1.399–7.027)	0.006	2.586 (1.111–6.021)	0.028
Pathologic tumor grade				
Low grade/ PUNLMP	Reference		Reference	
High grade	2.365 (0.936–5.973)	0.069	0.970 (0.310–3.035)	0.958
Adjuvant chemotherapy				
No	Reference		Reference	
Yes	0.324 (0.135–0.779)	0.012	0.331 (0.134–0.820)	0.017
Body mass index				
<Median	Reference			
≥Median	1.062 (0.442–2.552)	0.894		
Cardiovascular disease				
No	Reference			
Yes	0.918 (0.216–3.905)	0.908		
Hypertension				
No	Reference			
Yes	1.367 (0.588–3.175)	0.467		
Diabetes				
No	Reference			
Yes	0.803 (0.240–2.683)	0.721		
Drinking				
No	Reference			
Yes	0.543 (0.186–1.585)	0.264		
Smoking				
No	Reference			
Yes	0.432 (0.162–1.153)	0.094		
NLR				
≤2	Reference		Reference	
>2	3.866 (1.149–13.013)	0.029	2.211 (0.632–7.734)	0.214
PLR				
≤150	Reference			
>150	1.312 (0.591–2.912)	0.505		
Group				
ECV-lower	Reference		Reference	
ECV-higher	4.898 (1.929–12.435)	<0.001	4.093 (1.528–10.967)	0.005

ECV: extracellular volume fraction; SD: standard deviation; NLR: neutrophil-to-lymphocyte ratio; PLR: platelet-to-lymphocyte ratio; A/G ratio: albumin to globulin ratio; BMI: body mass index; PUNLMP: Papillary Urothelial Neoplasms of Low Malignant Potential.

**Figure 4.**
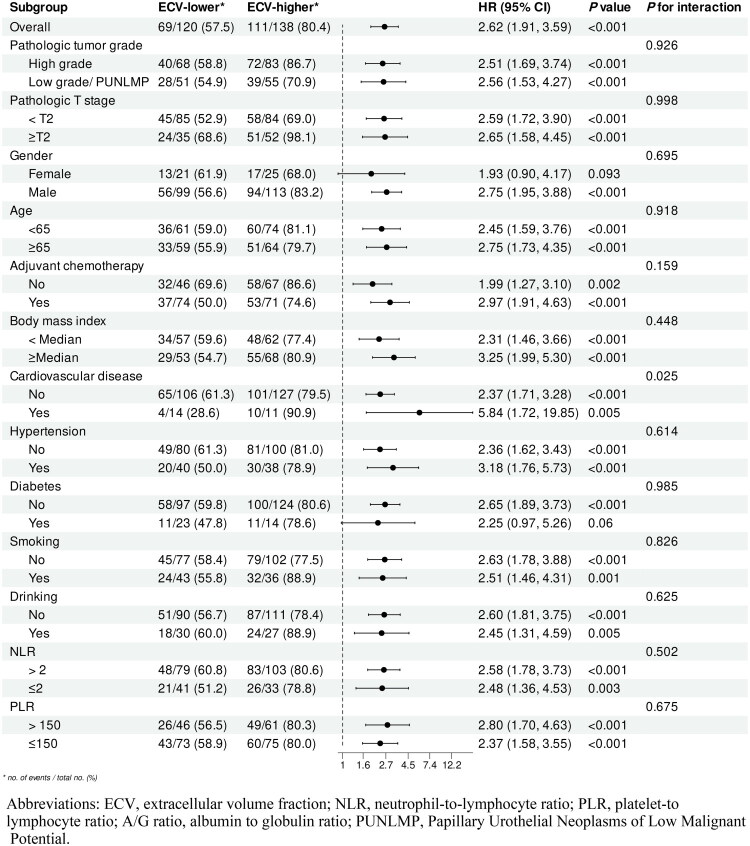
Subgroup analysis for RFS (ECV-higher vs ECV-lower).

**Figure 5.**
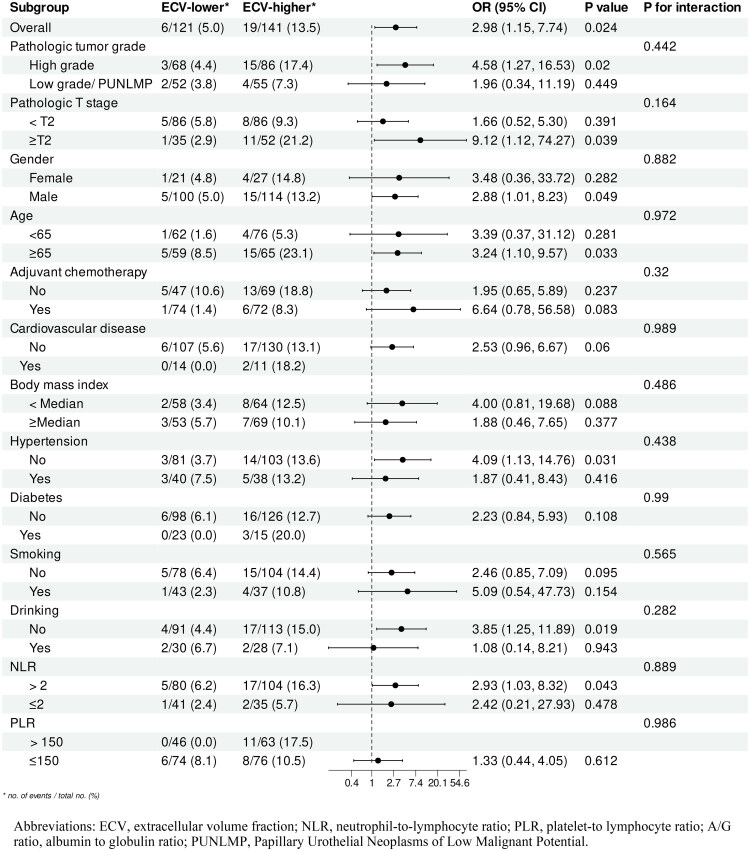
Subgroup analysis for OS (ECV-higher vs ECV-lower).

After PSM, we performed subgroup analysis according to baseline characteristics, and observed that the results for RFS and OS were relatively consistent. Each subgroup’s hazard ratios were obtained from univariate Cox models. In each subgroup of RFS (Figure 4), we found an interaction between cardiovascular disease and ECV, with ECV-higher group showing a higher risk in all subgroups, although the two subgroups of women, with diabetes, did not reach statistical differences. In the subgroup analysis of OS (Figure 5), the risk of short OS was higher in the ECV-higher group than in the lower ECV group, although there were no statistical differences in certain subgroups.

## Discussion

This study is the first to use ECV calculation to predict bladder cancer prognosis. Our results showed that compared to the high ECV group, patients in the low ECV group had longer RFS and OS. Preoperative ECV was an independent predictor of RFS and OS in bladder cancer patients (RFS, *p* < 0.001; OS, *p* = 0.002). Therefore, the application of tumor ECV derived from contrast-enhanced CT can serve as a non-invasive biomarker, assisting in the prognostic prediction for bladder cancer patients and providing valuable information for clinical decision-making.

ECV, as a noninvasive metric, offers actionable insights at diagnosis, enabling clinicians to identify high-risk patients who may benefit from neoadjuvant chemotherapy or aggressive surgical resection. Notably, our study utilized 3D tumor segmentation masks for extracellular volume (ECV) quantification, enhancing reproducibility relative to conventional 2D region-of-interest approaches while reducing sampling bias in heterogeneous tumor analysis. Furthermore, as a stable and quantitative parameter, it can independently account for various technical interferences and physiological variations [20]. The integration of ECV into prognostic models addresses a critical gap in BLCA management. In our study, compared to the high ECV group, patients in the low ECV group had longer RFS and OS. This is similar to previous findings in other cancers. Luo et al. [21] reported that in locally advanced colorectal cancer, the ECV in the pathological complete response (pCR) group was significantly lower than that in the non-pCR group, similarly, Chen et al. [22] found that in far-advanced gastric cancer, ECV in the pCR group was significantly lower than that in the non-pCR group, and the patients in the lower ECV group had a better tumor response, which is similar to the results of our study Gastric cancer is characterized by abundant desmoplastic stroma, and the fibrous stroma secreted by this tissue is associated with ECV. Several recent studies have also used ECV for oncological assessment, including identification of histological grading of renal clear cell carcinomas [23], histological classification of thymic epithelial tumors [24], and assessment of response and survival prediction of pancreatic tumors after chemotherapy [15,25]. This may be due to the higher the malignancy potential of the tumor, the higher the ECV score [23,24].

The association between ECV and tissue fibrosis has been well-established in studies on cardiovascular diseases, liver, and pancreas research [13,14,26]. The above observations are validated in the present study. One possible explanation is that activated tumor-associated fibroblasts (TAFs) in bladder cancer enhance immune evasion, thereby promoting tumor progression [27]. The prognostic significance of ECV likely stems from its ability to capture key features of the tumor microenvironment (TME). Tumor cells and tumor-associated fibroblasts, among others, can produce and deposit ECM components, and the extracellular matrix (ECM) undergoes extensive remodelling during tumor progression, affecting the tumor microenvironment, supporting tumor cell migration and invasion and promoting angiogenesis [28]. Elevated ECV may signify a fibrotic TME enriched with cancer-associated fibroblasts (CAFs) and immunosuppressive cells, which promote invasion and metastasis. For instance, CAFs secrete matrix metalloproteinases (MMPs) and growth factors (e.g. TGF-β) that degrade basement membranes and enhance tumor cell motility [29]. Similarly, a dense ECM can physically impede cytotoxic T lymphocytes, fostering immune evasion, a phenomenon observed in pancreatic [30]. Our findings align with studies linking high ECV to stromal activation in other malignancies, suggesting a conserved role for ECV as a surrogate for TME dysregulation. Numerous studies have confirmed that ECV scores are closely related to the quantity of connective tissue proliferative stroma [18,31]. In the context of bladder cancer, retrospective studies have shown that higher tumor stromal infiltration is associated with poorer survival prognosis [32–36]. Among these, CAFs are considered key players in BLCA tumor progression [37], this may be due to the fact that CAFs can secrete and remodel the fibrous stroma, which facilitates cancer invasion and metastasis [38]. Our correlation analysis showed a positive correlation between ECV and muscularis invasion, both before and after PSM, with BLCA patients exhibiting muscularis invasion having higher ECV scores, which was statistically significant and underscored its potential as a surrogate for tumor aggressiveness. Therefore, in BLCA, a higher ECV may indicate a higher tumor extracellular matrix, which is associated with poorer survival prognosis.

Our study has several limitations. First, the sample size was small and the study was conducted in a single center only. Therefore, larger sample sizes and multicenter validation will be important directions for future studies. Second, we used a 3–5 min delay period CT to measure tumor ECV scores. However, the time at which the equilibrium phase begins after the arterial phase scan is not fixed and the optimal time for estimating ECV using CT scanning after injection of iodinated contrast agent is uncertain. Luckily, Yoon et al. reported that a 3-minute equilibration period is a good compromise between clinical workflow and technical success [39]. Thirdly, different models of CT equipment were used for CT value measurements and thus for obtaining ECV scores. Although inherent variability between CT devices could theoretically be a confounding factor, consistent tube voltages and standardised contrast regimens were used across all devices. Given that the core objective of this study was to assess the relative rate of change in CT values, we believe that the impact of scanner differences on the final results is likely to be minimal. Finally, the hematocrit used to calculate ECV scores were obtained within 1 week before surgery. If hematocrit values could be measured on the same day as the CT scan, the results might be more reliable. In the future, we plan to collaborate with other centers to increase the sample size of the study by using different CT scanners from different manufacturers and to conduct further validation to enhance the generalizability of the findings.

In conclusion, this study establishes preoperative ECV as a robust, noninvasive biomarker for predicting outcomes in BLCA. Higher ECV values correlate with advanced pathological stage and independently portend poorer survival, offering a preoperative tool to guide personalized therapy. These findings advocate for the incorporation of ECV into multimodal risk assessment frameworks, potentially improving patient selection for intensified treatment regimens. Further research should explore the interplay between ECV, tumor biology, and therapeutic response to unlock its full clinical potential.

## Conclusions

Our study found that for BLCA patients, preoperative ECV was strongly associated with long-term prognostic prognosis. Future research should focus on elucidating the mechanisms underlying the observed association between ECV and prognosis. Additionally, integrating ECV assessments into routine follow-up may enhance the stratification of patients for tailored therapeutic strategies.

## Supplementary Material

Supplementary Materials.docx

## Data Availability

The datasets used and/or analyzed during the current study are available from the corresponding author [Lian Yang, e-mail yanglian@hust.edu.cn] upon reasonable request.
