# Supplementary material for: Extracellular volume fraction quantification by equilibrium contrast-enhanced CT via automated segmentation predicts survival outcomes in bladder cancer: a propensity score-matched study
Source: Ann Med. 2025 Jul 21;57(1):2534856. doi: 10.1080/07853890.2025.2534856 (PMC12284991; doi:10.1080/07853890.2025.2534856)
Supplement: Supplementary Materials.docx [file IANN_A_2534856_SM9019.docx]

**Supplementary Table 1.** **CT scanning protocol for ECV fraction**

| Parameter | Unenhanced CT | Arterial phase | Venous phase | Equilibrium phase |
| --- | --- | --- | --- | --- |
| Basic settings |  |  |  |  |
| Collimation (mm) | 64 x 0.625 / 32 x 1.2 / 48 x 1.2 | 64 x 0.625 / 32 x 1.2 / 48 x 1.2 | 64 x 0.625 / 32 x 1.2 / 48 x 1.2 | 64 x 0.625 / 32 x 1.2 / 48 x 1.2 |
| Rotation time (s) | 0.75/0.5 | 0.75/0.5 | 0.75/0.5 | 0.75/0.5 |
| Helical pitch | 0.891/0.8 | 0.891/0.8 | 0.891/0.8 | 0.891/0.8 |
| Slice thickness(mm) | 1.5/1.2 | 1.5/1.2 | 1.5/1.2 | 1.5/1.2 |
| Contrast-enhanced scanning | | | | |
| Dosage | | 300~350mg/ml | | |
| Rate (mL/s) |  | 2~3ml/s | | |
| Acquisition time (s)* |  | 30~35s | 60~80s |  |
| Coverage | Scanning from the superior pole of the kidney to the inferior border of the pubic symphysis | | | |

*Acquisition time means time interval from contrast injection to image acquisition

**Supplementary Table2. Cox Proportional Hazards Analyses for RFS before PSM analysis.**

| Parameter | Univariate analysis | | | | Multivariate analysis | |
| --- | --- | --- | --- | --- | --- | --- |
|  | Hazard ratio (95% CI) | | *P* value | | Hazard ratio (95% CI) | *P* value |
| Gender |  | |  | |  |  |
| Male | Reference | |  | |  |  |
| Female | 1.021 (0.697 - 1.495) | | 0.916 | |  |  |
| Age |  | |  | |  |  |
| < 65 | Reference | |  | |  |  |
| ≥ 65 | 0.848 (0.634 - 1.133) | | 0.265 | |  |  |
| Pathologic T stage |  | |  | |  |  |
| < T2 | Reference | |  | | Reference |  |
| ≥ T2 | 3.886 (2.843 – 5.312) | | < 0.001 | | 3.352 (2.296–4.893) | < 0.001 |
| Pathologic tumor grade |  | |  | |  |  |
| Low grade/ PUNLMP | Reference | |  | | Reference |  |
| High grade | 1.776 (1.313 – 2.404) | | < 0.001 | | 0.948 (0.656–1.370) | 0.775 |
| Adjuvant chemotherapy |  | |  | |  |  |
| No | Reference | |  | | Reference |  |
| Yes | 0.564 (0.420 – 0.758) | | < 0.001 | | 0.764 (0.559–1.044) | 0.091 |
| Body mass index |  | |  | |  |  |
| < Median | Reference | |  | |  |  |
| ≥ Median | 1.034 (0.765 - 1.397) | | 0.827 | |  |  |
| Cardiovascular disease |  | |  | |  |  |
| No | Reference | |  | |  |  |
| Yes | 0.685 (0.397 - 1.184) | | 0.175 | |  |  |
| Hypertension |  | |  | |  |  |
| No | Reference | |  | |  |  |
| Yes | 0.769 (0.555 - 1.065) | | 0.114 | |  |  |
| Diabetes |  | |  | |  |  |
| No | Reference | |  | |  |  |
| Yes | 0.763 (0.493 - 1.181) | | 0.225 | |  |  |
| Drinking |  | |  | |  |  |
| No | Reference | |  | |  |  |
| Yes | 0.995 (0.707 - 1.401) | | 0.976 | |  |  |
| Smoking |  | |  | |  |  |
| No | Reference | |  | |  |  |
| Yes | 0.870 (0.635 - 1.191) | | 0.384 | |  |  |
| NLR |  | |  | |  |  |
| ≤ 2 | Reference | |  | | Reference |  |
| > 2 | 1.391 (1.004 - 1.928) | | 0.047 | | 0.999 (0.684 - 1.458) | 0.995 |
| PLR |  |  | |  |  |  |
| ≤ 150 | Reference | |  | |  |  |
| > 150 | 1.316 (0.980 – 1.766) | | 0.068 | |  |  |
| Group |  | |  | |  |  |
| ECV-lower | Reference | |  | | Reference |  |
| ECV-higher | 3.772 (2.773 – 5.131) | | < 0.001 | | 3.338 (2.420 – 4.603) | < 0.001 |

Abbreviations: ECV, extracellular volume fraction; SD, standard deviation; NLR, neutrophil-to-lymphocyte ratio; PLR, platelet-to-lymphocyte ratio; A/G ratio, albumin to globulin ratio; BMI, body mass index; PUNLMP, Papillary Urothelial Neoplasms of Low Malignant Potential.

**Supplementary Table 3. Cox Proportional Hazards Analyses for OS before PSM analysis**

| Parameter | Univariate analysis | | | | Multivariate analysis | |
| --- | --- | --- | --- | --- | --- | --- |
|  | Hazard ratio (95% CI) | | *P* value | | Hazard ratio (95% CI) | *P* value |
| Gender |  | |  | |  |  |
| Male | Reference | |  | |  |  |
| Female | 1.024 (0.384 – 2.731) | | 0.962 | |  |  |
| Age |  | |  | |  |  |
| < 65 | Reference | |  | | Reference |  |
| ≥ 65 | 4.405 (1.653 – 11.742) | | 0.003 | | 8.119 (2.655–24.826) | < 0.001 |
| Pathologic T stage |  | |  | |  |  |
| < T2 | Reference | |  | | Reference |  |
| ≥ T2 | 3.762 (1.683 – 8.409) | | 0.001 | | 2.502 (0.947–6.607) | 0.064 |
| Pathologic tumor grade |  | |  | |  |  |
| Low grade/ PUNLMP | Reference | |  | | Reference |  |
| High grade | 2.804 (1.109 – 7.090) | | 0.029 | | 0.874 (0.282–2.705) | 0.815 |
| Adjuvant chemotherapy |  | |  | |  |  |
| No | Reference | |  | | Reference |  |
| Yes | 0.278 (0.115 – 0.669) | | 0.004 | | 0.309 (0.117–0.817) | 0.018 |
| Body mass index |  | |  | |  |  |
| < Median | Reference | |  | |  |  |
| ≥ Median | 0.998 (0.415 – 2.398) | | 0.996 | |  |  |
| Cardiovascular disease |  | |  | |  |  |
| No | Reference | |  | |  |  |
| Yes | 0.816 (0.192 – 3.470) | | 0.783 | |  |  |
| Hypertension |  | |  | |  |  |
| No | Reference | |  | |  |  |
| Yes | 1.263 (0.543 – 2.935) | | 0.588 | |  |  |
| Diabetes |  | |  | |  |  |
| No | Reference | |  | |  |  |
| Yes | 0.820 (0.245 – 2.740) | | 0.747 | |  |  |
| Drinking |  | |  | |  |  |
| No | Reference | |  | |  |  |
| Yes | 0.543 (0.186 - 1.586) | | 0.264 | |  |  |
| Smoking |  | |  | |  |  |
| No | Reference | |  | | Reference |  |
| Yes | 0.416 (0.156 - 1.111) | | 0.080 | | 0.565 (0.203 - 1.572) | 0.274 |
| NLR |  | |  | |  |  |
| ≤ 2 | Reference | |  | | Reference |  |
| > 2 | 4.848 (1.437 – 16.351) | | 0.011 | | 2.290 (0.645 – 8.128) | 0.200 |
| PLR |  |  | |  |  |  |
| ≤ 150 | Reference | |  | |  |  |
| > 150 | 1.558 (0.700 – 3.468) | | 0.277 | |  |  |
| Group |  | |  | |  |  |
| ECV-lower | Reference | |  | | Reference |  |
| ECV-higher | 6.083 (2.401 – 15.412) | | < 0.001 | | 5.914 (2.025 – 17.273) | 0.001 |

Abbreviations: ECV, extracellular volume fraction; SD, standard deviation; NLR, neutrophil-to-lymphocyte ratio; PLR, platelet-to-lymphocyte ratio; A/G ratio, albumin to globulin ratio; BMI, body mass index; PUNLMP, Papillary Urothelial Neoplasms of Low Malignant Potential.

**Supplementary Figure 1. Multi-phase CT image sequences of a 77-year-old male patient with non-muscle-invasive bladder cancer (NMIBC).**


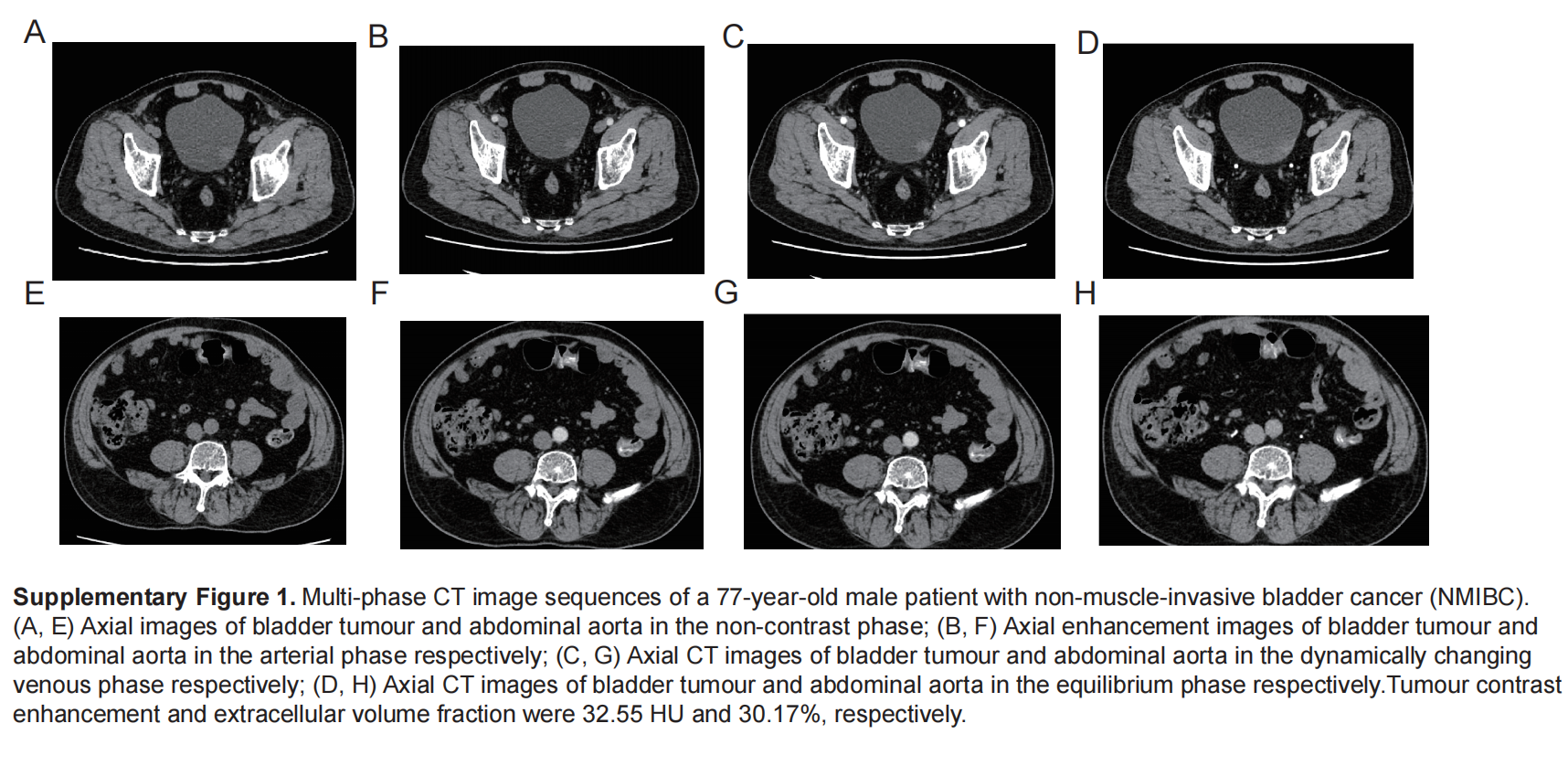


**Supplementary Figure 2. A 77-year-old man with bladder cancer (NMIBC) .**


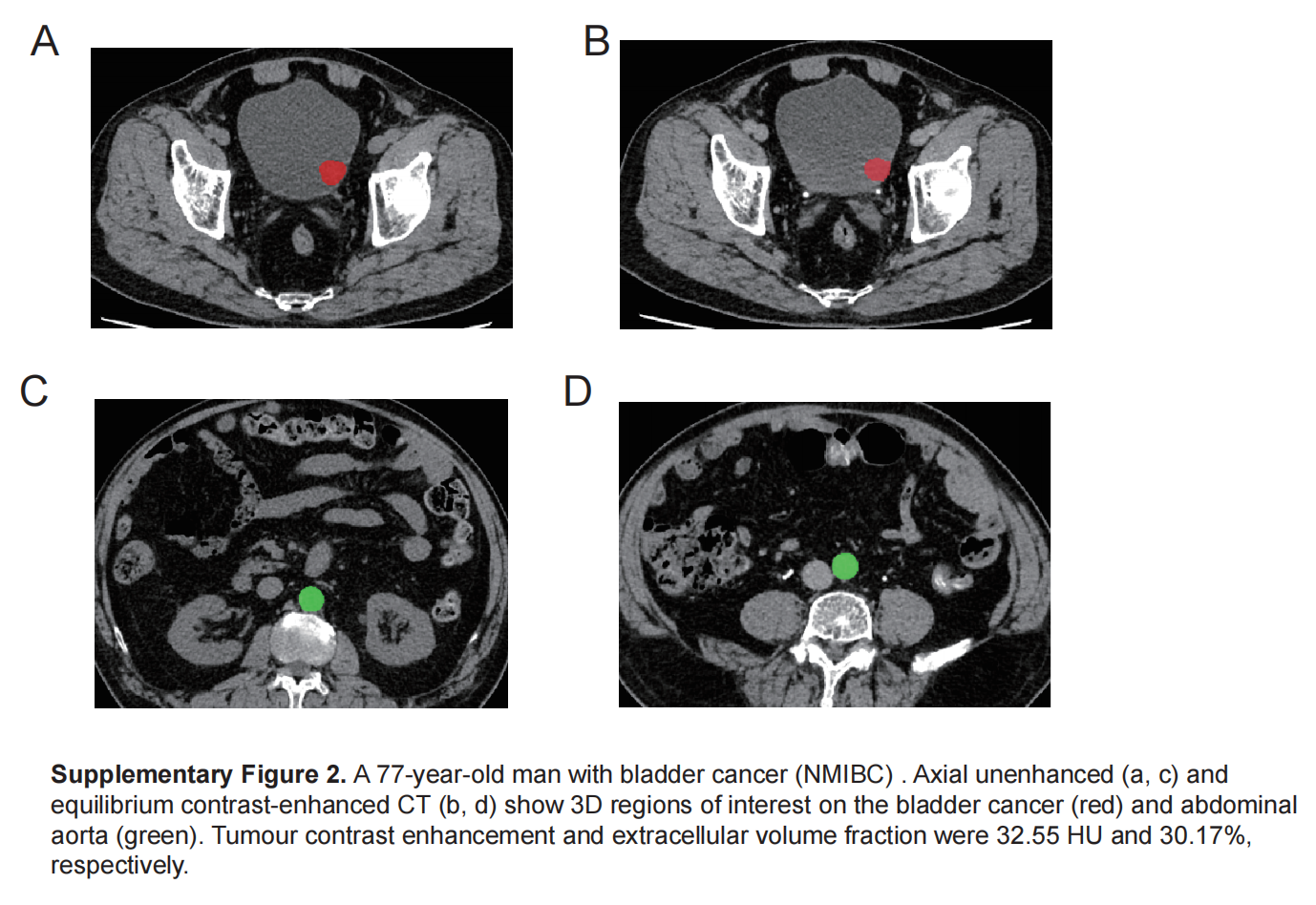


**Supplementary Figure 3. Concordance Assessment: Manual Segmentation and nnU-Net Automated Delineation by Bland-Altman Analysis.**


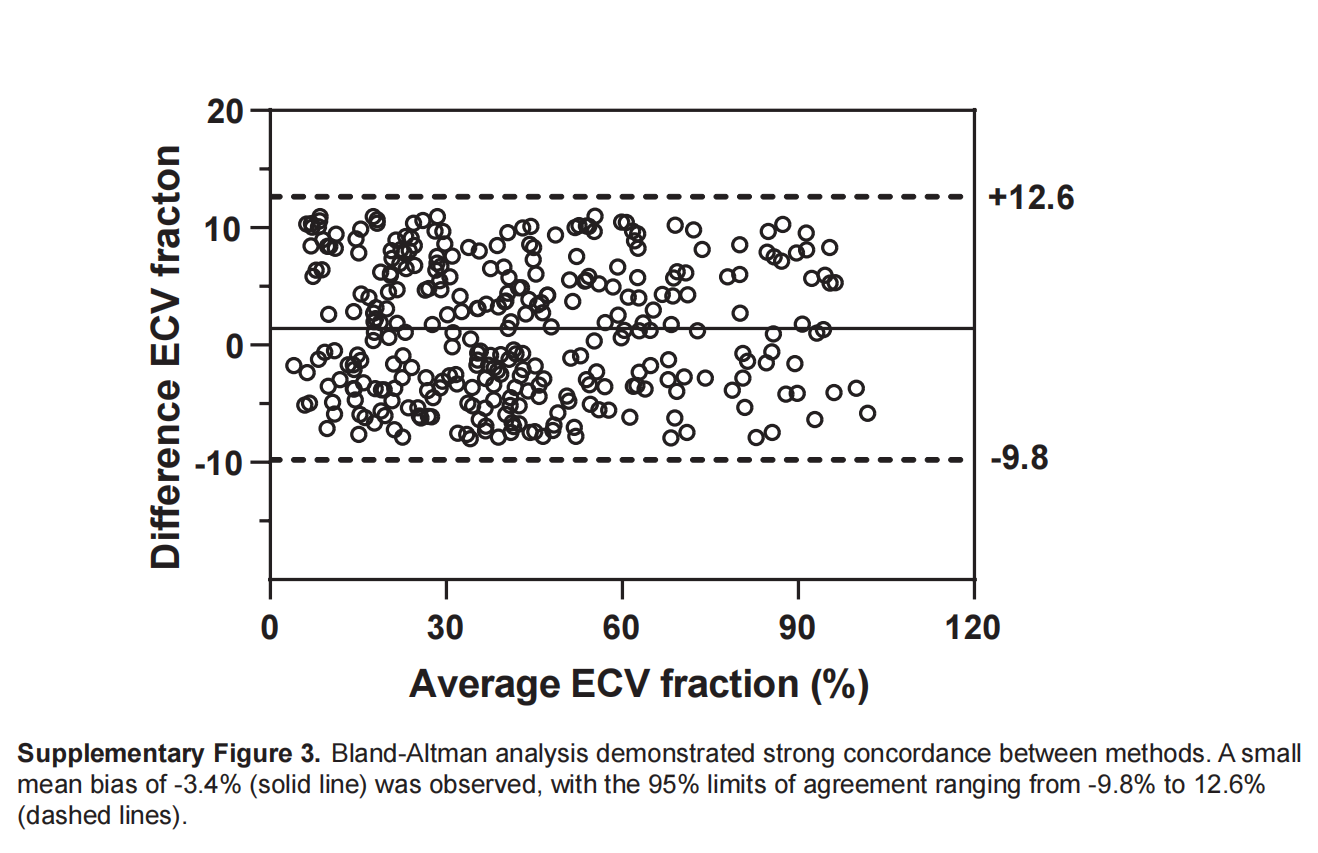
Abbreviations: ECV, extracellular volume fraction.

**Supplementary Figure 4. The optimal cutoff value for ECV classification were established by the survminer software**


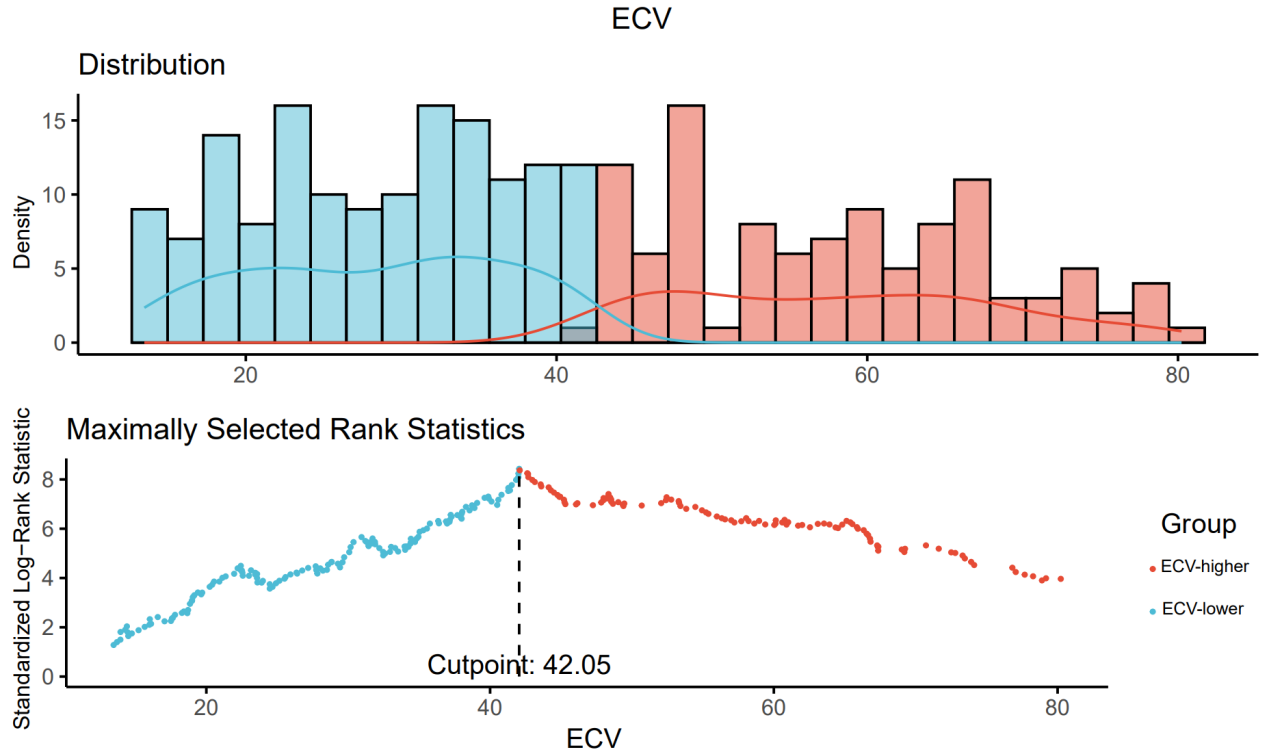


Abbreviations: ECV, extracellular volume fraction;

The cut-off point for changes in extracellular volume fraction was determined using the maximally selected log-rank statistic. The optimal threshold for ECV was found to be 42.05%.
